# Joint Union Meeting of the New Jersey and Pennsylvania State Dental Societies

**Published:** 1891-08

**Authors:** 


### JOINT UNION MEETING OF THE NEW JERSEY AND PENNSYLVANIA STATE DENTAL SOCIETIES.

The meeting of these Societies at Asbury Park, N. J., July, 15–17, was a most successful gathering. The number in attendance was very large, many from distant sections being doubtless attracted by the place of meeting as well as by the excellent programme arranged by the joint committee.

Several valuable papers were read, but the recasting of old ideas was noticeably prominent here as elsewhere. A serious drawback was the reading of papers by proxy. Writers of essays should endeavor to be present and read their productions, for no matter how well they may be presented, and these were exceedingly well done, the life of the paper is measurably destroyed by the process.

The plan of conducting meetings, originating in England, of having one or more to lead in the discussion of a paper, was adopted here, and in great measure destroyed that spontaneity of discussion so valuable in all such gatherings.

The New Jersey Society accepted an invitation from the Pennsylvania State Dental Society to meet with them in joint convention next year. No place was selected, but it was anticipated that the meeting would be held at Cresson, Pa.

